# Genetically identified *Salmonella typhimurium* outbreak linked to a rural Butcher’s Shop, February–March 2018, North East England

**DOI:** 10.1017/S0950268824001547

**Published:** 2024-12-05

**Authors:** Nicola Love, Anaïs Painset, Heather Aird, Hayley Coleman, Shirley Sorrell, Claire Stoker, Petra Manley, Deborah Wilson

**Affiliations:** 1 Field Service, UK Health Security Agency, Northeast and Yorkshire & Humber, Newcastle, UK; 2 National Institute for Health Research Health Protection Research Unit (NIHR HPRU) in Gastrointestinal Infections, University of Liverpool, Liverpool, UK; 3 Evaluation and Epidemiological Sciences division, UK Health Security Agency, UK; 4 Gastrointestinal Bacteria Reference Unit, UK Health Security Agency, London, UK; 5 UK Health Security Agency, Food, Water and Environmental Microbiology Services, York, UK; 6 County Durham and Darlington NHS Foundation Trust, UK; 7 Environmental Health, Durham County Council, Durham, UK; 8 North East Health Protection Team, UK Health Security Agency, Newcastle, UK

**Keywords:** Salmonella, whole genome sequencing, case-control study, outbreak, Epidemiology

## Abstract

Between February and April 2018, *Salmonella typhimurium* within a unique 5-single nucleotide polymorphism (SNP) address was isolated from 28 cases with links to a small rural area of Northeast England, with five cases prospectively identified by whole genome sequencing (WGS). Infections had a severe clinical picture with ten cases hospitalized (36%), two cases with invasive disease, and two deaths reported. Interviews determined that 24 cases (86%) had been exposed to a local independent butcher’s shop (Butcher A).

A case-control study using controls recruited by systematic digit dialling established that cases were 68 times more likely to have consumed cooked meat from Butcher A (Adjusted OR 68.1; 95% CI: 1.9–2387.6; *P* = 0.02). *Salmonella typhimurium* genetically highly related to 28 of the outbreak cases was also isolated from a sample of cooked meat on sale in the premises.

Epidemiological and microbiological investigations suggest this outbreak was likely associated with the consumption of ready-to-eat foods supplied by the implicated butcher. A relatively large number of cases were involved despite the rurality of the food business, with cases resident across the Northeast and Yorkshire identified using WGS, demonstrating the benefit of timely sequencing information to community outbreak investigations.

## Introduction

*Salmonella Typhimurium* is a commonly reported gastrointestinal pathogen, causing a range of symptoms including asymptomatic infections, gastroenteritis, and infrequently systemic disease. Death occurs in less than 1% of cases, although this can be considerably higher in outbreaks associated with elderly or immunocompromised individuals [1]. The majority of cases are self-limiting with symptoms generally resolving within 7 days. Transmission is predominantly via the consumption of contaminated food sources, most frequently undercooked or unpasteurized animal products [2, 3]. Cross-contamination of hands, cooking surfaces/equipment and ready-to-eat (RTE) food can also occur when handling contaminated raw meat, and infections resulting from contact with an infected person or animal via the faeco-oral route are also common.

Since April 2014, the Gastrointestinal Bacteria Reference Unit (GBRU), UK Health Security Agency (UKHSA), formerly Public Health England has routinely performed whole genome sequencing (WGS) on all salmonella isolates in England, providing a single nucleotide polymorphism (SNP) address, which allows for greater discrimination between strains and the determination of phylogenetic relationships [4]. WGS is increasingly used in outbreak investigations to determine common exposures between seemingly unrelated cases and to strengthen temporal and geographical associations, with isolates separated by 5 SNP differences indicative of a common source [5].

The increasing use of WGS allows for improved strain discrimination supporting outbreak investigation. It has been used to prospectively detect multi-country outbreaks associated with a single food exposure [6], in the investigation of prolonged outbreaks linked to the unusual exposure of a drainage system at a food premises [7], to identify clusters without apparent epidemiological links [8] and to identify when localized outbreaks are part of a more widespread international cluster [9]. Here, we describe the benefit of using prospective sequencing information as a method of case finding in community outbreaks where a likely source of infection is known.

## Outbreak detection

On 14 February 2018, the North East Health Protection Team (HPT) became aware of an exceedance in laboratory-confirmed salmonella cases in Area A, a small relatively rural area of 20000 people, through an alert triggered in the North East’s infectious disease surveillance system for the week commencing 12 February 2018 (9 cases reported compared with an expected weekly number of 1). Concurrently, environmental health officers (EHOs) identified three cases in the restricted geographical area who reported consuming food from the same butcher, Butcher A. Butcher A was a longstanding family-run enterprise in a close-knit rural community supplying raw and ready-to-eat (RTE) foods to the public and to several local businesses. A suspected outbreak was declared, and a multi-disciplinary outbreak control team (OCT) convened on 16 February 2018 to investigate further and implement control measures.

## Methods

### Descriptive epidemiology

Passive case finding used local primary care providers, routine laboratory notifications and routinely notified WGS results. Active case finding was performed by alerting local microbiological laboratories and General Practitioners (GPs). In addition, a proactive media statement was released on 21 February advising residents within the local area to seek medical attention if unwell. WGS-linked primary confirmed cases were defined in the final case definition as a person with a laboratory-confirmed salmonella infection with the SNP address 1.1.1.124.3255.4475.% (5 SNP cluster t5.4475), a sample date on or after 22 January 2018, and a link to Butcher A or Area A. Possible cases were defined as a person with diarrhoea on or after 22 January 2018, with a link to food supplied by Butcher A or Area A but without laboratory-confirmed salmonella. Secondary cases were defined as a person with a laboratory-confirmed salmonella infection (t5.4475), and a sample date on or after 22 January 2018, who had been in close contact with a confirmed or possible primary case and had symptom onset at least three days after the primary case.

### Case-control study

An unmatched case-control study was undertaken to test the null hypothesis that illness caused by *Salmonella typhimurium* (t5.4475) was not associated with the consumption of foods supplied by Butcher A. Outbreak cases were excluded from the case-control study if detected through staff screening; deceased or a household contact of a deceased case; were unable to recall exposure information; were reported to the HPT after control recruitment commenced on 1 March 2018. Control recruitment was attempted by case nomination, voluntary recruitment from GP practices and systematic digit dialling. Controls were defined as adults (> = 18 years of age) residents in Area A, who had not developed diarrhoea and/or vomiting on or after 1st February 2018, had not travelled abroad or had close contact with a symptomatic individual in the seven days prior to the interview.

Case and control interviews were conducted by telephone between 27 February and 28 March 2018 using tailored questionnaires capturing information about diarrhoeal illness and potential risk exposures from 1st February 2018 onward. Exposures included consumption of raw and RTE items from 8 local butcher’s shops, specifying 9 types of raw meat items and 12 types of RTE items, Responses were entered into SelectSurvey and analyzed using Stata v13. Univariable odds ratios (OR) were calculated to test the association between illness and the purchase and consumption of raw and RTE foods from local butchers. Confounding exposures were determined by stratification using the Mantel–Haenszel method. Multivariable regression was performed by exact logistic regression, with demographic variables and all food exposures with a *P*-value of 0.2 or less added to the model using a forward stepwise approach. The resulting models were compared by likelihood-ratio testing with significant exposures retained in the model.

### Clinical microbiology and phylogeny

Stool samples were requested from any suspected salmonella case and all staff employed by Butcher A (*n* = 18). All positive isolates were sent to the UKHSA GBRU for confirmation and identification of the species and sequence type (ST). Whole genome sequencing (WGS) was performed. Quality trimmed short reads were mapped against the *Salmonella typhimurium* reference genome (AE006468) and high-quality variant positions were extracted. Hierarchical single-linkage clustering was performed on the pairwise SNP distance matrix between isolates resulting in an ‘SNP address’ that describes the cluster membership of isolates [4]. SNP addresses were generated for each isolate and a phylogenetic tree was constructed from all UKHSA isolates at the 25 SNPs level of the suspected outbreak SNP address.

### Environmental investigations

EHOs visited Butcher A on multiple occasions to investigate food hygiene practices and obtained formal food and environmental samples on 15 and 20 February 2018. Additionally, EHOs visited two local premises supplied by Butcher A to sample food items. All environmental samples were submitted to the UKHSA Food, Water and Environmental (FWE) microbiology laboratory in York.

## Results

**Figure 1.**
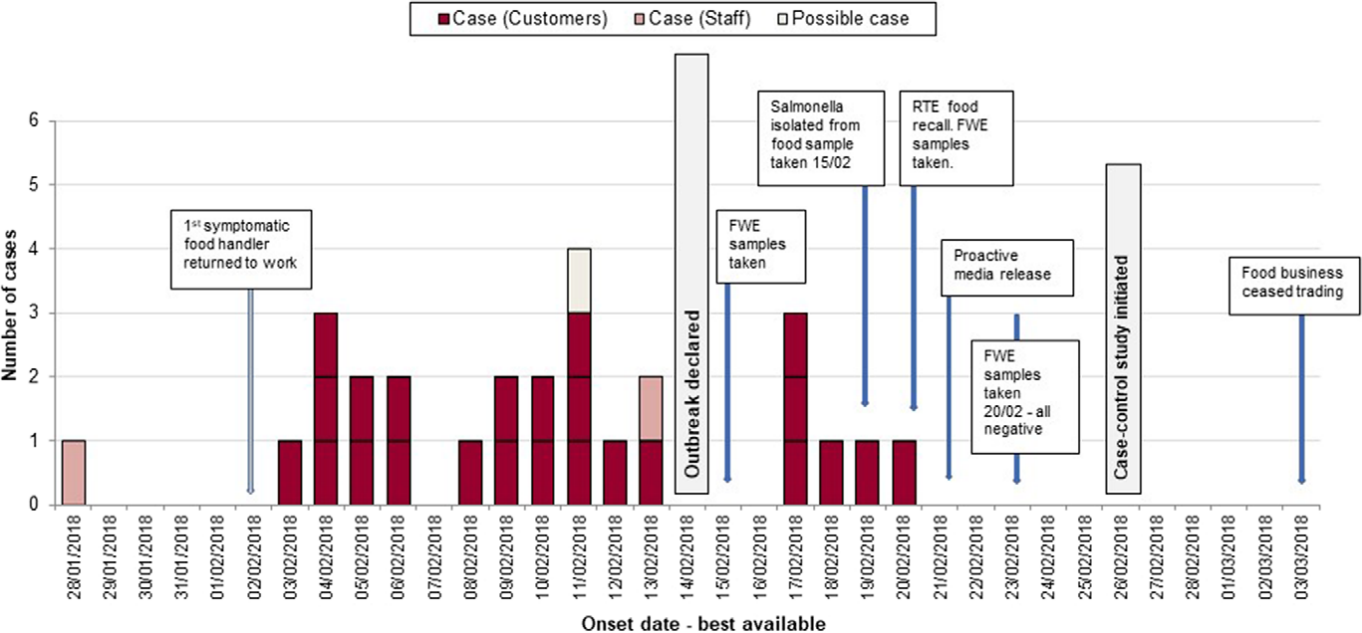
Epidemic curve for cases of Salmonella linked to Butcher A or Area A including confirmed and possible cases by onset date, January to April 2018. *N* = 28. The onset date was estimated for one possible primary case and one staff member. The onset date was unknown for one case. One staff member was asymptomatic.

Between 9 February 2018 and 5 April 2018, the North East HPT was notified of 30 *Salmonella typhimurium* ST 34 cases (t5.4475) in individuals with links to Area A. Twenty-eight cases met the primary confirmed case definition, with symptom onset dates ranging from 28 January (estimated for the earliest case based on healthcare attendance) to 21 February 2018 (Figure 1), suggestive of prolonged exposure to a common source. The earliest confirmed onset date was 4 February 2018. Infections were severe, with ten cases hospitalized (36%); two cases diagnosed with invasive disease and two deaths where salmonella was a contributing factor were reported (7%). Two secondary cases and one primary possible case were also identified.

Those affected were generally older adults (median age: 60.5 years (range 10–75 years), males (64% male, *n* = 18), and residents in Northeast England (93%, *n* = 26). Seven cases resided outside of Area A, including two cases from outside the Northeast region; however, all cases had visited Area A during their incubation period. One case (non-staff case) residing outside of Area A (case 27) reported international travel in addition to travel to Area A, no other cases reported travel. Twenty-four cases (82%) had a confirmed link to Butcher A, including three staff members identified through screening.

Cases reported a range of exposures; 22 cases (including two staff members) consumed food purchased from the shop; one case was a staff member who denied consuming food from the premises; one case consumed food from a buffet provided by Butcher A and two cases consumed RTE food at a local business supplied by Butcher A. Of the four cases without a clear link to the retailer, two were not normally resident in the area but had visited during their incubation period and one case was suspected to have visited Butcher A but salmonella was diagnosed posthumously.

### Case-control study

Fourteen confirmed cases met the criteria for inclusion in the case-control study (referred to as study cases). Study cases and non-study cases did not differ significantly by age, sex or area of residence (data not shown). The mean illness duration was 14.2 days (range at the time of interview: 5–24 days; *n* = 14); which was longer than commonly observed for salmonella infections. Of note, on follow-up, one case reported symptoms and positive microbiology 122 days after initial infection. All 14 study cases reported diarrhoea (100%), 11 reported experiencing fever (79%) and 5 reported vomiting (36%).

Twenty-three controls were included in the study (1.6 controls per case). Case nomination of controls was poor (*n* = 2), and recruitment by voluntary commitment from GP practices was unfeasible due to a combination of resource constraints and data protection concerns from GP providers. A systematic digit dialling method was therefore used for recruitment (*n* = 21). Recruited controls were significantly older than cases (67 years, IQR 59–77 years vs. 58 years, IQR 37–68 years; Wilcoxon rank-sum *P* = 0.03), with cases non-significantly more likely to be male (64% vs. 35%; Pearson Chi^2^ (χ2) = 3.05; *P* = 0.08). The proportion of cases and controls living more than two miles from Butcher A were comparable (43% vs. 39%; χ^2^ = 0.05; *P* = 0.82).

Thirteen cases (93%) and ten controls (44%) reported purchasing raw or RTE items from any independent butcher in the local area. Cases purchased food from only one retailer (Butcher A), while controls purchased food from six different retailers including Butcher A. The odds of purchasing food from Butcher A were 37 times higher for cases (95%CI: 3.6–1653.7; *P* = <0.001; Table 1). All cases (*n* = 14) and ten controls (44%) reported consuming products from a local butcher.

**Table 1.** Case-control study results. Association between illness and consumption of raw or RTE food, from local butchers. Northeast England, February to April 2018

		Case			Control						Multivariable^¥^		
Exposure	Food item	Total	Exposed	%	Total	Exposed	%	OR	95% CI	*P*-value	aOR	95% CI	p-value
Raw^a^ – Any Butcher		14	6	42.9	23	6	26.1	2.1	0.4–10.8	0.470			
Raw^a^ – Butcher A		14	6	42.9	23	3	13.0	5	0.8–36.9	0.057			
	Any raw processed	14	4	28.6	23	2	8.7	4.2	0.5–51.6	0.174			
	Any raw joint	14	4	28.6	23	3	13.0	2.7	0.4–21.3	0.390			
Raw^a^ – another butcher		14	0	0.0	23	4	17.4	0	0.0–1.5	0.276			
RTE – Any Butcher		14	13^c^	92.9	23	10^b^	43.5	36.8	3.6–1653.7	<0.001			
RTE – Butcher A		14	14^c^	100.0	23	6	26.1	-	[8.8-.]	<0.001			
	Any cooked meat	14	11	78.6	23	4	17.4	17.4	2.6–132.1	<0.001	68.1	1.9–2387.6	0.020
	Any ham item	14	9	64.3	23	4	17.4	8.6	1.5–52.9	0.004			
	Any sandwich filling	14	7	50.0	23	2	8.7	10.5	1.4–117.4	0.005	14.4	0.6–338.2	0.098
	Any hot sandwich	14	2	14.3	23	0	0.0	-	[0.9 -.]	0.062			
	Any pork item	14	5	35.7	23	4	17.4	2.6	0.4–16.5	0.208			
	Any pie	14	3	21.4	23	3	13.0	1.8	0.2–15.8	0.502			
	Any cold sandwich	14	3	21.4	23	3	13.0	1.8	0.2–15.8	0.502			
	Any savoury pastry	14	2	14.3	23	2	8.7	1.8	0.1–26.7	0.595			
RTE – another butcher		14	0	0.0	23	6	26.1	0	0.0–0.9	0.645			

a Raw meat refers to purchase rather than consumption as cases and controls were frequently unable to recall exact dates of consumption.

b Three controls visited two butcher’s shops and are included twice in stratification but once in overall total.

c One case did not report purchasing food from a local butcher, but was known to have consumed cooked meats at a second premises supplied by Butcher A.

Butcher A was the only retailer to have a statistically significant association between consumption of any food item and being a case (*P* = <0.001). The association between the purchase of raw meat from Butcher A and being a case (OR 5, 95% CI: 0.8–37.0) was not statistically significant and was reduced by stratification by the consumption of RTE items from Butcher A (adjusted OR 0.8, 95% CI: 0.1–5.1, percentage change = −85.0%; *P* = 0.77). A significant association was observed between the consumption of RTE items from Butcher A and becoming a case (95%CI: [8.8 –.]; *P* = <0.001).

Consumption of any cooked meat item (OR 17.4; 95%CI: 2.6–132.1; *P* = <0.001), any ham item (cooked meat/prepared sandwich; OR 8.6; 95% CI: 1.4–117.4; *P* = 0.004), and any non-meat sandwich filler (OR 10.5; 95% CI: 1.4–117.4; *P* = 0.01) were significant exposures identified by univariate analysis. Multivariable analysis identified cooked meat as the only significant food exposure, with the odds of eating cooked meat from Butcher A 68 times higher for cases after adjusting for sex, age and area of residence (95% CI: 1.9–2387.6; *P* = 0.02; Table 1). However, it is important to note that as only ~80% of cases ate cooked meat items from Butcher A, the consumption of cooked meat was not sufficient to explain illness in all cases exposed to Butcher A.

### Clinical microbiology and phylogeny

**Figure 2.**
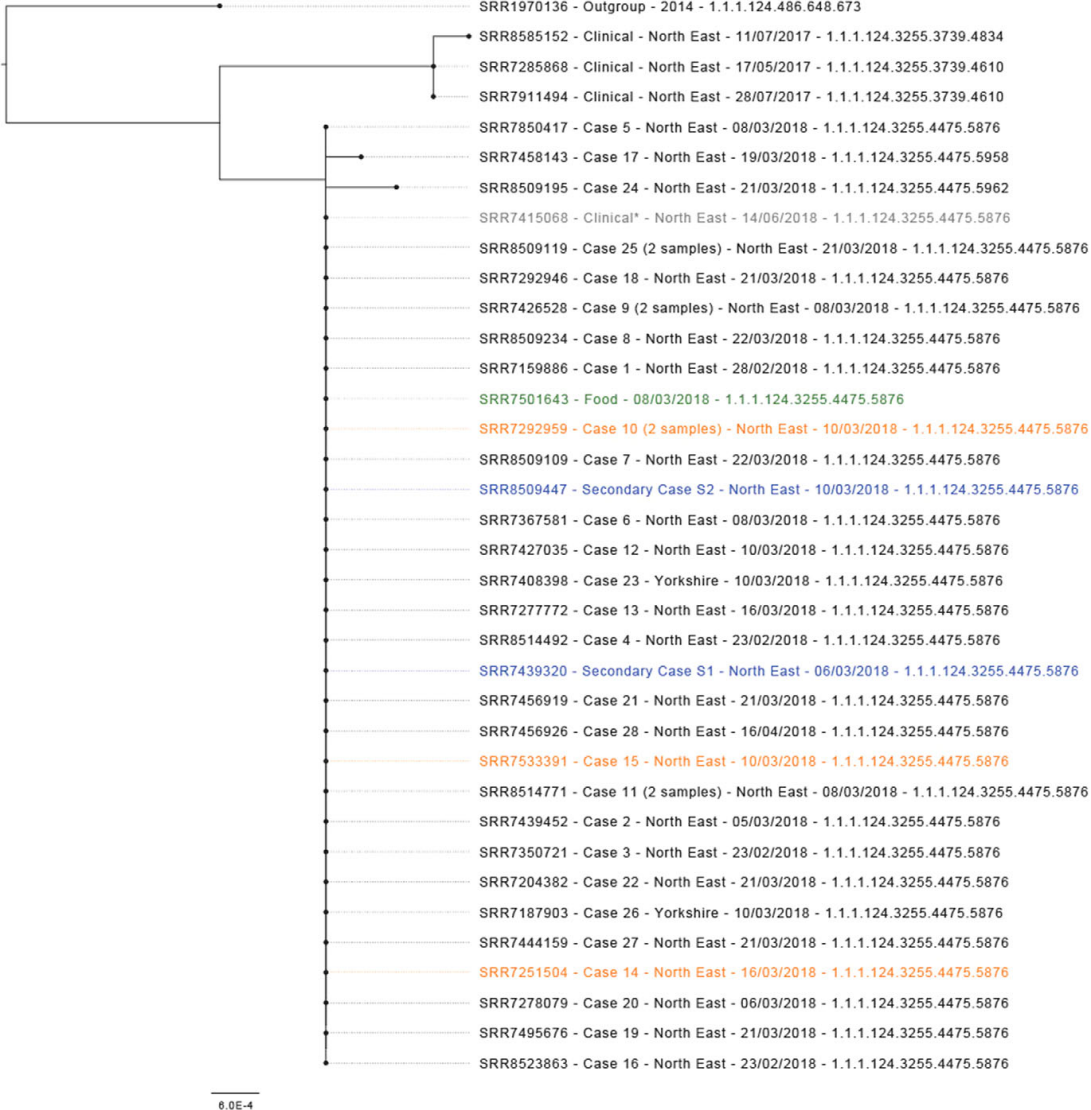
Maximum-likelihood phylogenetic tree (*N* = 36) representing outbreak strains with context at 10 SNP (t10) level. Isolates in blue are secondary cases, isolates in orange are staff member cases, the isolate in green is a food sample and the isolate in grey and labelled as clinical* clustered genetically but was not part of the outbreak.

*Salmonella Typhimurium* ST 34 was isolated from 30 case samples (28 primary confirmed and 2 secondary), including from a blood culture sample and post-mortem faecal and splenic swabs (*n* = 2). Isolates from 26 primary confirmed cases and two secondary cases, including three staff member cases, were found to have an identical SNP address (1.1.1.124.3255.4475.5876; Figure 2). Two further isolates identified from primary confirmed cases differed by one SNP (1.1.1.124.3255.4475.5958) and two SNPs (1.1.1.124.3255.4475.5962). Five outbreak cases were detected through WGS, including two individuals living outside of the Northeast region, one North East case initially believed to be foreign travel associated and one North East case without recorded exposures. Phylogenetic analysis showed closely related isolates. The closest outgroup was >15 SNPs, with the outbreak strain unique to the Northeast of England. Three genetically similar isolates were reported in the Northeast between May and July 2017, separated from the outbreak cluster by approximately 10 SNPs. A further case within the 0 SNP cluster was identified in a Northeast resident with the onset of symptoms in May 2018, after the completion of the outbreak investigation. This case was from the same geographical area as the outbreak cases, but no epidemiological link was identified. The Case had been hospitalized due to unrelated health issues for several weeks prior to the sample being taken and subsequently died, therefore exposure information was limited. As of December 2023, there have been no further cases within the 5 SNP cluster reported.

### Environmental

All staff employed by Butcher A (*n* = 18) denied recent illness, but three salmonella-positive staff members were identified through screening (one asymptomatic, one subsequently became symptomatic, and one symptomatic in late January who reportedly did not work while symptomatic). All staff members reported consuming products purchased from the premises, of which two consumed RTE food items.

Previous routine environmental health officer inspections of the premises were positive with adequate separation observed between raw and RTE areas. Environmental investigations carried out during the outbreak raised concerns about access to adequate handwashing facilities, poor staff hand hygiene, inadequate cleaning procedures and the risk of cross-contamination from raw to RTE foods.

All environmental swabs taken from the front serving counter and raw and RTE preparation areas were negative for salmonella and for hygiene markers (*n* = 16; Table 2). Raw and RTE foods were sampled and salmonella genetically highly related to that of 28 clinical cases was isolated from cooked pork on sale on 15 February 2018. Samples of RTE cooked pork, cooked ham and pease pudding were also found to have high aerobic colony counts, high *Escherichia coli* counts and high Enterobacterales counts, suggesting poor hygiene practices.

**Table 2.** Food samples taken at Butcher A, on 15 and 20 February 2018 during the outbreak that occurred in Northeast England from February to April 2018

Date of sample	Item sampled	Salmonella isolated	Aerobic colony count (cfu/g)	Other organisms (cfu/g)
15th February 2018	Cooked ham from chiller display	No	98636	Enterobacterales^a^ (250)
	Stuffing from the chiller display	No	320909	
	Pease pudding from the chiller display	No	5004545	Enterobacterales^a^ (295)
	Roast pork from the chiller display	**Yes**	18000000	*Escherichia coli* (9450); Enterobacterales^a^ (54545)
20th February 2018	Steak pie from the display cabinet	No	<7000	
	Sliced cooked ham from chilled display	No	<7000	
	Sliced cooked tongue from chilled display	No	<7000	
	Sliced cooked belly pork from the display	No	<7000	
	Sliced cooked beef from chilled display	No	<7000	
	Cooked pork from refrigerator	No	<7000	
	Cooked beef from refrigerator	No	<7000	

a Reported as Enterobacteriaceae.

### Interventions

EHOs provided the food business operator with advice on deep cleaning and hand hygiene. Following the detection of salmonella in a sample of cooked meat, all businesses supplied by Butcher A were advised on 20 February to discard any RTE food in the supply chain. On 21 February, following the diagnosis of salmonella in a food handler, a proactive media statement was released advising the public to discard any RTE foods from Butcher A. The food business operator voluntarily ceased trading indefinitely on 3 March 2018.

## Discussion

The described outbreak resulted in a relatively large number of severe infections requiring rapid investigation and the prompt implementation of control measures. Despite the rurality of the implicated food business, cases were resident across the Northeast and outside the region, although all had visited Area A prior to onset. Epidemiological and microbiological investigations suggested that *Salmonella* cases within a 5 SNP outbreak cluster (1.1.1.124.3255.4475.%) were linked and that the outbreak was therefore likely associated with exposure to a common source.

The results of the case-control study supported the conclusion that illness was associated with the consumption of RTE foods supplied by Butcher A. No significant association was observed between consuming food items from other local independent butchers and developing salmonella. Univariable analyses identified several RTE items associated with increased odds of illness, with the consumption of cooked meat being the only food item found to be significantly associated with illness when adjusted for demographic factors. However, it is important to note that the consumption of cooked meat from Butcher A did not explain illness in all cases and that the consumption of other food items could have resulted in illness, but the small sample size meant the study was not powered to determine these associations.

*Salmonella* is commonly found in raw meat but should be eradicated with appropriate cooking. The presence of salmonella on cooked meat found on sale in the premises and the increased odds of illness following the consumption of cooked meat suggest that either (I) cooked meat on sale in the premises was not adequately cooked, (II) there had been cross-contamination between raw and cooked meat on sale in the premises or (III) there had been cross-contamination between a shedding food handler and cooked meat on sale in the premises. The OCT agreed that the evidence gathered strongly supports the hypothesis of post-cooking cross-contamination of RTE products.

Three staff members tested positive for salmonella with one staff member reporting gastrointestinal illness prior to the outbreak. All members of staff reported consuming products from the premises. As the earliest staff member case did not have a stool sample taken while symptomatic, we cannot rule out other causative agents. Therefore, while we cannot rule out contamination by a shedding staff member, all staff members also reported consuming products from the premises. While it is possible that contamination from shedding staff member(s) handling RTE products was a potential mechanism of transmission, particularly given environmental health officer concerns around hand hygiene, we do not have sufficient evidence to confirm this.

This study shows the potential for WGS to be used prospectively for case finding during outbreak investigations. Five outbreak cases without a geographical link were detected specifically through WGS and would not have been linked through traditional epidemiological methods alone. Furthermore, WGS was able to confirm that several geographically and temporally associated cases without links to Butcher A were part of the same outbreak and it was subsequently established that Butcher A supplied RTE items to a second local business where both cases had eaten.

While this outbreak investigation was strengthened by microbiological, epidemiological and environmental evidence, there were several limitations. Interviews of several cases and all controls occurred after media coverage of the outbreak and may have introduced information bias. Furthermore, the retailer was well regarded within the local community and negative perceptions of the outbreak investigation within the community and on social media may also have biased findings. Indeed, several cases and controls were aware of the premises under investigation and defended the retailer during telephone interviews. During the outbreak investigation, the food business operator ceased trading with immediate effect. As a result, several controls were interviewed after the closure of the premises. While significant associations were found between the implicated butcher and illness, the relatively small number of cases and controls included in the study means that confidence intervals are large and should be interpreted with some caution.

The case-control study was undertaken after Butcher A had been identified as the potential source of the outbreak. The epidemiological evidence gathered was important for several reasons: to ensure that the OCT had identified the correct source of the cases as if it had not been Butcher A there may have been a different uncontrolled source of salmonella in the community; to provide additional epidemiological evidence to support any possible legal action; to exclude concerns that as Butcher A was used by many people in the area that this was why it had been identified as the cause of the outbreak rather than because of a causal association; to strengthen the evidence that food from Butcher A was the source of the outbreak in the context that the local community was denying that Butcher A was the source of the outbreak.

In conclusion, this study described an outbreak of *Salmonella Typhimurium* (1.1.1.124.3255.4475.%) linked to the consumption of foods supplied by a local independent butcher. A strong association with the purchase and consumption of RTE foods, particularly cooked meats was demonstrated, and a sample of cooked meat taken from the premises of Butcher A tested positive for the outbreak strain. Furthermore, as some cases were prospectively identified through the use of WGS this study demonstrates the benefits of timely sequencing information in community outbreak investigations.

### Data sharing

FASTQ sequences were deposited in the NCBI Short Read Archive under the BioProject PRJNA248792 (https://www.ncbi.nlm.nih.gov/bioproject/?term=248792). SRR accession numbers for this outbreak are shown on the phylogenetic tree.

## References

[1] Tam CC, Rodrigues LC, Viviani L, Dodds JP, Evans MR, Hunter PR, et al. (2012) Longitudinal study of infectious intestinal disease in the UK (IID2 study): Incidence in the community and presenting to general practice. Gut 61(1), 69–77. 21708822 10.1136/gut.2011.238386 PMC3230829

[2] Milnes AS, Stewart I, Clifton-Hadley FA, Davies RH, Newell DG, Sayers AR, et al. (2008) Intestinal carriage of verocytotoxigenic Escherichia coli O157, Salmonella, thermophilic Campylobacter and Yersinia enterocolitica, in cattle, sheep and pigs at slaughter in Great Britain during 2003. Epidemiology & Infection 136(6), 739–751. 17655782 10.1017/S0950268807009223 PMC2870870

[3] Little CL, Richardson JF, Owen RJ, de Pinna E, Threlfall EJ. (2008) Campylobacter and Salmonella in raw red meats in the United Kingdom: Prevalence, characterization and antimicrobial resistance pattern, 2003–2005. Food Microbiology 25(3), 538–543. 18355680 10.1016/j.fm.2008.01.001

[4] Ashton PM, Nair S, Peters TM, Bale JA, Powell DG, Painset A, et al. (2016) Identification of Salmonella for public health surveillance using whole genome sequencing. PeerJ 4, e1752. 27069781 10.7717/peerj.1752 PMC4824889

[5] Waldram A, Dolan G, Ashton PM, Jenkins C, Dallman TJ (2018) Epidemiological analysis of Salmonella clusters identified by whole genome sequencing, England and Wales 2014. Food Microbiology 71:39–45. 29366467 10.1016/j.fm.2017.02.012

[6] Inns T, Ashton PM, Herrera-Leon S, Lighthill J, Foulkes S, Jombart T, et al. (2017) Prospective use of whole genome sequencing (WGS) detected a multi-country outbreak of Salmonella Enteritidis. Epidemiology & Infection 145(2), 289–298. 27780484 10.1017/S0950268816001941 PMC9507544

[7] Mair-Jenkins J, Borges-Stewart R, Harbour C, Cox-Rogers J, Dallman T, Ashton P, Johnston R, Modha D, Monk P, Puleston R (2017) Investigation using whole genome sequencing of a prolonged restaurant outbreak of Salmonella Typhimurium linked to the building drainage system, England, February 2015 to March 2016. Euro Surveill 22(49), 17–00037. doi: 10.2807/1560-7917.ES.2017.22.49.17-00037. PMID: 29233257; PMCID: PMC5727591. PMC5727591 29233257

[8] Rathnayake IU, Graham RMA, Bayliss J, Staples M, Micalizzi G, Ariotti L, Cover L, Heron B, Graham T, Stafford R, Rubenach S, D’Addona A, Jennison AV (2023) Implementation of routine genomic surveillance provided insights into a locally acquired outbreak caused by a rare clade of Salmonella enterica serovar Enteritidis in Queensland, Australia. Microbial Genomics 9(7):mgen001059. doi: 10.1099/mgen.0.001059. PMID: 37459172; PMCID: PMC10438802 37459172 PMC10438802

[9] Benson HE, Reeve L, Findlater L, Vusirikala A, Pietzsch M, Olufon O, Matthews E, Hoban A, Painset A; Incident Management Team; Balasegaram S, Larkin L, Weir S, Heinsbroek E (2023) Local Salmonella Enteritidis restaurant outbreak investigation in England provides further evidence for eggs as source in widespread international cluster, March to April 2023. Eurosurveillance 28(27):2300309. doi: 10.2807/1560-7917.ES.2023.28.27.2300309. PMID: 37410382; PMCID: PMC10370042. 37410382 PMC10370042

